# Selection and Characterization of a DNA Aptamer Specifically Targeting Human HECT Ubiquitin Ligase WWP1

**DOI:** 10.3390/ijms19030763

**Published:** 2018-03-07

**Authors:** Wesley O. Tucker, Andrew B. Kinghorn, Lewis A. Fraser, Yee-Wai Cheung, Julian A. Tanner

**Affiliations:** School of Biomedical Sciences, Li Ka Shing Faculty of Medicine, The University of Hong Kong, 21 Sassoon Road, Hong Kong, China; wtucker@hku.hk (W.O.T.); kinghorn@hku.hk (A.B.K.); lewis-fraser@hku.hk (L.A.F.); cheungw@hku.hk (Y.-W.C.)

**Keywords:** aptamer, SELEX, ubiquitin ligase, WWP1, targeted drug delivery

## Abstract

Nucleic acid aptamers hold promise as therapeutic tools for specific, tailored inhibition of protein targets with several advantages when compared to small molecules or antibodies. Nuclear WW domain containing E3 ubiquitin ligase 1 (WWP1) ubiquitin ligase poly-ubiquitinates Runt-related transcription factor 2 (Runx2), a key transcription factor associated with osteoblast differentiation. Since WWP1 and an adapter known as Schnurri-3 are negative regulators of osteoblast function, the disruption of this complex has the potential to increase bone deposition for osteoporosis therapy. Here, we develop new DNA aptamers that bind and inhibit WWP1 then investigate efficacy in an osteoblastic cell culture. DNA aptamers were selected against three different truncations of the HECT domain of WWP1. Aptamers which bind specifically to a C-lobe HECT domain truncation were observed to enrich during the selection procedure. One particular DNA aptamer termed C3A was further evaluated for its ability to bind WWP1 and inhibit its ubiquitination activity. C3A showed a low µM binding affinity to WWP1 and was observed to be a non-competitive inhibitor of WWP1 HECT ubiquitin ligase activity. When SaOS-2 osteoblastic cells were treated with C3A, partial localization to the nucleus was observed. The C3A aptamer was also demonstrated to specifically promote extracellular mineralization in cell culture experiments. The C3A aptamer has potential for further development as a novel osteoporosis therapeutic strategy. Our results demonstrate that aptamer-mediated inhibition of protein ubiquitination can be a novel therapeutic strategy.

## 1. Introduction

In mammalian bone, the osteoblast is exclusively responsible for the deposition of bone matrix in the form of hydroxyapatite crystals together with various structural proteins [1]. The osteoblast lineage is largely controlled by Runx2 (Runt–related transcription factor 2/CBFA1/PEBP2αA), often considered the “master regulator” of osteoblast differentiation [2]. The importance of Runx2 is illustrated by observations that Runx2 Runx2 null mice cannot produce mineralized bone due to a lack of early stage osteoblasts [3]. The Nedd4 family HECT (‘Homologous to the E6-AP Carboxyl Terminus’) domain E3 ligase WWP1, in complex with an adapter Schnurri-3 (Shn3), ubiquitinates Runx2 thus leading to its degradation and reduction of osteoblast-mediated bone matrix synthesis [2]. WWP1 and Shn3 proteins negatively regulate Runx2 at the protein level, hence are promising targets to stimulate osteoblast differentiation, and by extension, higher bone mass in osteoporosis patients [4,5]. Human WWP1 was discovered in 1997 and contains an independently active HECT domain (Homologous to the E6-AP carboxyl terminus) which has its crystal structure solved [6,7,8]. WWP1 is structurally and functionally characterized, is readily “expressible” in *E. coli*, and inhibition of WWP1 to maintain Runx2 levels is a promising therapeutic strategy to stimulate osteoblast function. 

Nucleic acid aptamers are short, single-stranded nucleic acid chains evolved to bind specifically to a given target. Riboswitches can be considered as natural non-coding RNA aptamers which regulate gene expression upon binding a metabolic target [9]. Since 1990, aptamers have been created in the laboratory using a process known as Systematic Evolution of Ligands by Exponential Enrichment (SELEX) [10,11]. First, a large pool of random sequences (~10^15^) is exposed to a target, washed to exclude non-binding species, and eluted to recover binding species. Second, the pool is PCR amplified by having included 3’ and 5’ primer binding regions. Finally, the sequences are enriched by repeating the process successively while introducing increased stringency (i.e., more rigorous washing conditions and counter-selections of related targets). The SELEX iterations are continued until enriched pools of homologous sequences are revealed, which then hold the potential to be tightly binding and specific [10,11]. Aptamers often bind to their targets with *K*_D_ values in the µM to nM range with aptamer affinity maturation developed to increase affinity and specificity [12]. Aptamers have secondary structure including hairpins, loops, pseudoknots, triplexes, and quadruplexes [13,14,15]. SELEX can be applied to a wide variety of targets ranging from small molecules to cells [16,17]. Due to these complexities and features, aptamers are sometimes compared and contrasted with both antibodies and small molecules for use as therapeutics [18,19], diagnostics [20,21,22], and laboratory tools [23]. Importantly, recent work has demonstrated methods that lower the cost of single stranded oligonucleotide synthesis by several orders of magnitude [24].

Aptamers have traditionally focused on extracellular targets. However, polynucleotides such as aptamers can enter the intracellular space by either: (1) extraneous means such as liposomes, polymers, viruses, microinjection, electroporation, particle bombardment, calcium phosphate precipitation and ultrasound, or by (2) inherent mechanisms such as endocytosis or pinocytosis [25,26,27,28]. Using cationic reagents, aptamers can be transfected as is commonly practiced with plasmids. Naked DNA, without the company of reagents, does enter the cell to some degree in spite of electrostatic repulsion, as was demonstrated when internalized plasmids were found to express proteins [29]. Uptake depends both on temperature and oligonucleotide length [30]. It has also been found that two putative cell surface receptors are specific to oligonucleotides generally [31]. More recently, aptamers have been conjugated to moieties to bind an internalizing receptor, or may be selected to bind the internalizing receptor themselves [26,32]. Approximately 19,000 DNA molecules can be internalized by one cell once cationic lipoplexes have fused with the anionic plasma membrane [33]. Aptamers can also be transcribed directly in the cell, this type of aptamer has been coined an “intramer” [34,35,36].

Previously, our group selected aptamers which bind and inhibit the activity of the extracellular protein sclerostin, which is a known negative regulator of bone formation [19]. Here, we investigate an alternative intracellular strategy where the intracellular protein WWP1 is targeted with a DNA aptamer. Herein we select and characterize DNA aptamers against the HECT domain of WWP1. We investigate aptamer binding to its target, specific inhibition of its function, observe localization, and evaluate efficacy in SaOS-2 osteoblastic cells.

## 2. Results

### 2.1. Strategy to Select DNA Aptamers against HECT Domain of WWP1

As it is not possible to express the full length WWP1 protein in *E. coli*, truncations of the smaller yet active HECT domain were used as the targets for aptamer selection. The HECT domain has two lobes: the N-lobe is involved in E2 binding and the C-lobe is important in ubiquitination [8]. Rotation around a hinge between the two lobes is critical to WWP1 ubiquitin ligation activity [8], hence the HECT domain is central to WWP1 function. 

**Figure 1:**
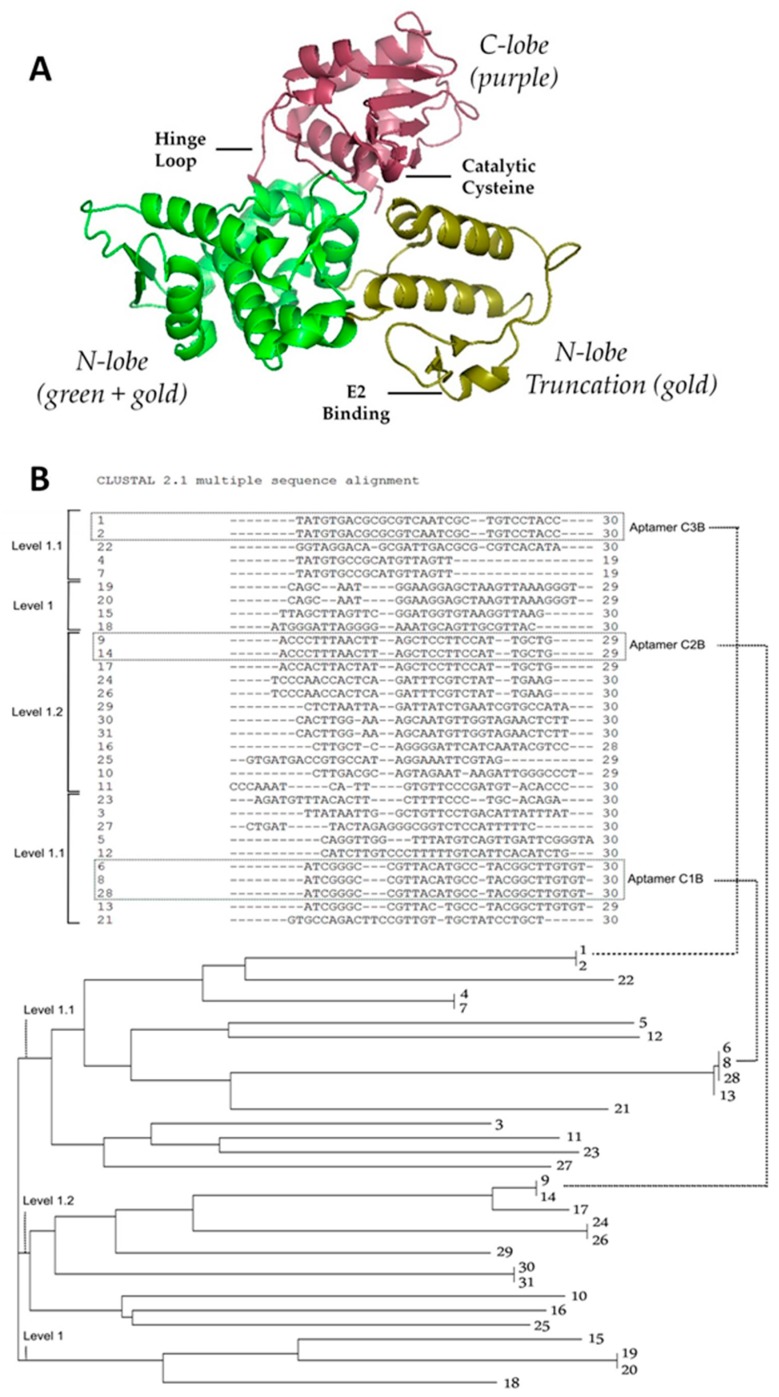
Structure of WWP1 HECT domain target and selected pool. (**A**) WWP1 HECT structure as generated by pyMOL with functionally important regions labeled; (**B**) Variable regions of the enriched pool for C-lobe with the three most abundant groups of identical sequences are boxed (C1–C3 where B indicates without constant regions), and phylogenetic tree of sequenced aptamers showing inter familial relationships.

As shown in Figure 1A, we expressed and purified three WWP1 truncations for use as targets in our aptamer selections: the entire HECT domain, the N-lobe of the HECT domain and the C-lobe of the HECT domain. 

To begin, we used the entire HECT domain for the first 8 rounds of DNA aptamer selection. Important to its inhibition, HECT domain requires a flexible hinge loop, the binding of an E2 adapter, and a catalytic cysteine residue for activity (Figure 1A). We then proceeded to lobe-specific selections to target particular functional regions more specifically. To further increase specificity as well as stringency, C-lobe selections included N-lobe counter selections, N-lobe selections included C-lobe counter selections, and HECT selections included ubiquitin as a general protein counter selection. During the sequencing of pools throughout the process, the C-lobe pools proved to enrich promisingly, while HECT and N-lobe selections did not enrich. This indicated that DNA aptamers had been selected in the initial rounds that likely bind to the C-lobe of the HECT domain. 

We performed 4 further SELEX lobe-specific rounds (after the 8 round HECT domain pre-enrichment). Cloning and sequencing identified 31 aptamer sequences against the C-lobe truncation (Figure 1B), annotated as C*#*A when constant region is included and C*#*B without. The relationships between the variable regions is somewhat scattered—C3 is more closely related to C1 while C2 is completely unrelated. These three sequences were chosen to move forward in the project due to their higher copy number in sequencing data.

**Figure 2:**
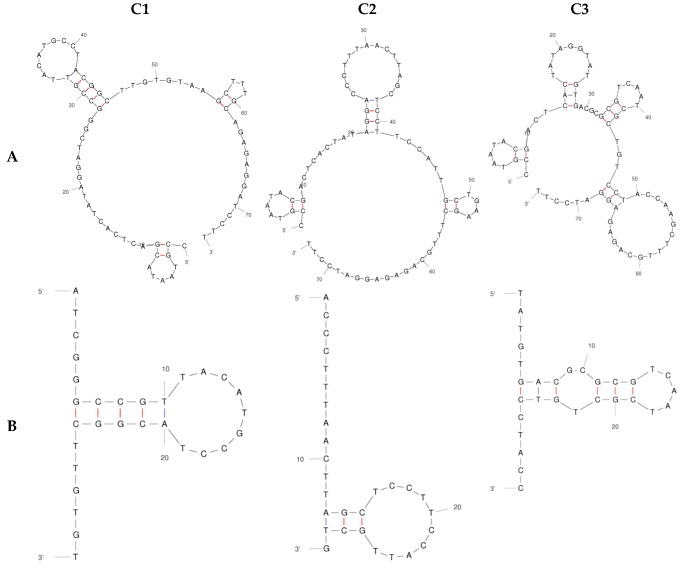
Predicted 2D secondary structures for C-lobe aptamers using mFold software comparing full length (**A**) with variable region (**B**) simulated at 150 mM NaCl and 25 °C.

To compare the predicted secondary structures of the three aptamers, mFold software (version 2.3, The RNA Institute, State University of New York at Albany, Albany, NY, USA) was used (Figure 2). The structure of the variable region mirrors that of its full-length counterpart for aptamer C1, but not for C2 and C3; possibly indicating that full length is required for C2 and C3 binding (Figure 2). Experiments were therefore carried out subsequently with constant regions included (indicated by the A suffix). 

### 2.2. Determination of Binding Affinity of DNA Aptamers Binding to HECT Domain

**Figure 3:**
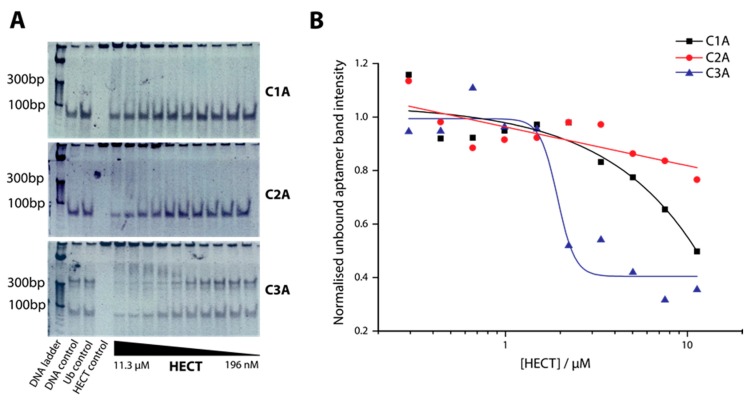
Determination of *K*_D_ for aptamers using electrophoretic mobility shift assay (EMSA) for aptamers C1A, C2A, and C3A. (**A**) 10% PAGE gels showing bound (upper bands) versus unbound (lower bands) aptamer; (**B**) Plot of HECT concentration versus normalized band intensity (unbound) with a one-site binding fit from representative data in Figure 3A.

We next investigated the relative affinity of the DNA aptamers for the protein target relative to control. To demonstrate qualitative binding of the C-lobe aptamers to our recombinant HECT, Electrophoretic Mobility Shift Assay (EMSA) was employed (Figure 3). EMSA is a hallmark method for characterization of protein-nucleic acid binding. However, EMSA has shortcomings such as complexes differing in stability depending on the gel medium and secondary binding confusing the magnitude of the binding of interest. Thus, we view EMSA here as a relative and semi-quantitative characterization of binding. The full-length aptamers C1A–C3A (the A indicating inclusion of the constant flanking region) were incubated with a range of HECT concentrations then analyzed by PAGE electrophoresis (Figure 3A), and the resultant band intensities were plotted (Figure 3B). Aptamer bound to protein remains at the top of the gel, while free aptamer migrates normally. Of the three C-lobe aptamers, C3A showed the strongest binding affinity. We observed that C3A shows a multimeric band above its monomer band also, which may imply a multimer in equilibrium with the monomer (74 bp). A *K*_D_ roughly in the low µM range was estimated for aptamer C3A, while other C-lobe aptamers and controls did not produce an inflection point with which to base a determination but with clearly weaker binding. Therefore, in later experiments we focused on aptamer C3A as the most promising of the three aptamer candidates.

### 2.3. Determination of C-Lobe DNA Aptamer-Mediated Inhibition of Ubiquitination Activity of HECT Domain 

**Figure 4:**
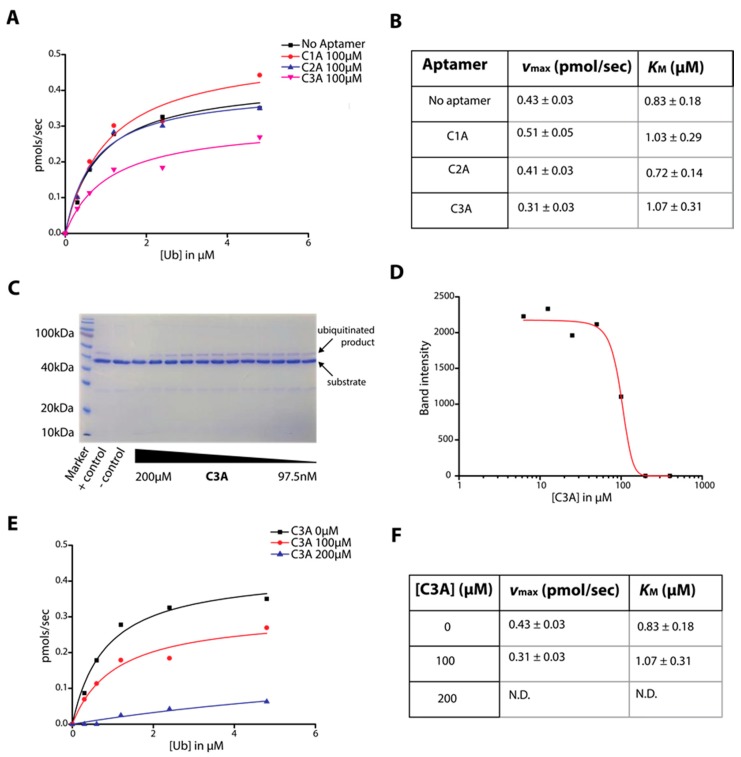
Aptamer mediated inhibition of HECT ubiquitination. (**A**) Rate of reaction product formation plotted against ubiquitin concentration (Michaelis-Menten) for aptamers C1A–C3A to compare inhibition rates between aptamers from representative gel data. (**B**) Resultant *V*_max_ and *K*_M_ values from the Michaelis-Menten plot. (**C**) 12% SDS-PAGE gel showing the reaction product over increasing C3A concentrations; (**D**) Logarithmic plot of C3A aptamer concentration to band intensity as measured to determine IC_50_ from representative gel data (**E**) Michaelis-Menten plot of two different C3A concentrations for determination of mode of inhibition from representative gel data. (**F**) Summary of *V*_max_ and *K*_M_ values tabulated for the two C3A concentrations.

The HECT domain’s ability to self-ubiquitinate with the requirement of an E1 and E2 protein in vitro provides an approach to observe the kinetics of HECT domain activity. When the HECT domain is ubiquitinated, the HECT domain-ubiquitin conjugate is observed by SDS-PAGE appearing several kilodaltons above HECT and can be used for quantitation (Figure 4C). We used this assay to generate Michaelis-Menten curves between aptamers C1A–C3A which implicates aptamer C3A as the strongest inhibitor of the three (Figure 4A).

The resulting *v_max_* and *K_M_* are tabulated in Figure 4B and reconfirms aptamer C3A as the strongest inhibitor. This result is also consistent with our observations of aptamer binding in Figure 3. Figure 4C shows the ubiquitination assay on an example gel over a series of aptamer C3A concentrations, where substrate and reaction product are labeled (residual ubiquitin (~10 kD) and E2 (~10 kD) can also be seen). The plot of C3A concentration to band intensity of reaction product (Figure 4D) determines an IC_50_ of approximately 100 µM. To determine mode of inhibition, two different C3A concentrations were tested (100 µM and 200 µM) while varying the concentration of ubiquitin substrate to determine *K_M_* and *v_max_* as shown in Figure 4E,F. The presence of the inhibitor caused a significant reduction in the apparent *v*_max_ while the apparent *K_M_* remained similar. These results imply that the aptamer acts as a non-competitive inhibitor of ubiquitination.

### 2.4. Cellular Localization, Runx2 Levels, and Bone Mineralization in Saos-2 Osteoblastic Cells

**Figure 5:**
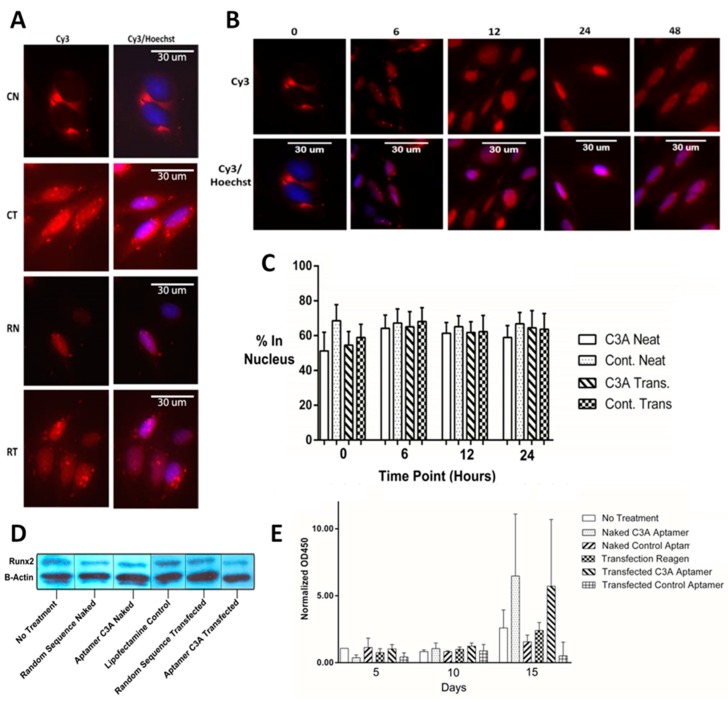
Aptamer C3A localization, and effect on Runx2 levels and bone mineralization in SaOS-2 cells. (**A**) Aptamer (Cy3) nucleus (Hoechst) overlays where: RN—random sequence control without transfection, RT—random sequence control with transfection, CN—aptamer C3A without transfection, and CT—aptamer C3A with transfection; (**B**) Aptamer and nucleus overlays over time points in hours; (**C**) Quantitative assessment of nuclear localization using MetaMorph™ software for transfected or naked (labeled as “NEAT”) aptamer C3A and random sequence control; (**D**) Western Blot of Runx2 performed on SaOS-2 cell extracts; (**E**) Quantitation of extracellular matrix deposition over time using Alizarin Red assay.

Fixed and stained SaOS-2 cells were imaged after the transfection process to investigate differences in localization. These stained images were used to compare aptamer C3A and control, both with and without transfection reagent as shown in Figure 5A. Although the images are overexposed, we were able to gain information about the relative abundance of aptamer in the cytoplasm versus nucleus. In this representative example, aptamer C3A without transfection (CN) appears to enter the cell, but appears mostly proximal to the nucleus, whereas aptamer C3A with transfection (CT) seems to localize both in the nucleus and cytosol.

After determining that aptamer enters the cells without transfection reagent, we compared localization over time by incubating aptamer C3Awith SaOS-2 cells without transfection reagent over 5 time points (Figure 5B). All aptamer samples appear to enter the cell and the images depict migration towards the nucleus over time. Percent nuclear localization over time was quantified by image intensity where hundreds of cells were batch processed using MetaMorph™ software (64-bit version, Molecular Devices Corp., Sunnyvale, CA, USA) for aptamer C3A and control, with and without transfection (Figure 5C). Whether or not transfection reagent was used, aptamers appear to enter the cell and localize partially in both the cytoplasm and nucleus to some degree. There are examples where all aptamers are exclusively in the cytoplasm for a period of time, but these results show that for most cells, a proportion (>50%) of the aptamers end up in the nucleus quickly after exposure. To see if treatment with aptamer C3A had an effect on Runx2 protein expression levels in SaOS-2 cell extracts, a western blot was performed (Figure 5D). Data were consistent with a slight reduction of Runx2 levels when cells were transfected with C3A relative to controls (Figure 5D). To determine if aptamer C3A influenced osteoblast ability to deposit extracellular matrix, SaOS-2 cells were subjected to an Alizarin Red assay to quantify calcific deposition (Figure 5E). At a timepoint 15 days after transfection it was clear that both the naked C3A aptamer and the transfected C3A aptamer were able to increase the rate of calcific deposition of the cells relative to control aptamers (Figure 5E). This data would be consistent with an observation that the C3A aptamer is able to enter the cell even in the absence of the transfection reagent and increase calcific extracellular matrix deposition. We also investigated the influence of aptamer C3A on apoptosis and observed that aptamer C3A promoted apoptosis significantly more than a random sequence control (Figure S1). 

## 3. Discussion

Aptamer selections were begun with an entire HECT domain ‘pre-selection’. Subsequent selections against N-lobe, C-lobe and HECT pool showed enrichment of Aptamers only against C-lobe. This indicated that the C-lobe likely had selected tight-binding aptamers early in the selection process. We observed that it was necessary to retain the flanking constant regions in the C-lobe aptamers. Two classic examples of aptamers for which the PCR constant regions were not necessary for binding are pegaptanib and the anti-thrombin aptamer, but others have been reported where constant regions were required for binding [37,38]. We surmise that the necessity of flanking regions in an aptamer varies from selection to selection.

Concerning our WWP1 truncation targets themselves (Figure 1), some structural features are relevant. First, the C-lobe portion of the related E3 ubiquitin ligase UbR5 has its crystal structure solved and was found to be active [39]. Nevertheless, we assumed our C-lobe truncation was unlikely to have activity considering previous reports had shown that the N-lobe is necessary for the ubiquitination reaction [8]. Our best C-lobe targeting aptamer C3A, which had been pre-selected against HECT domain, was shown to specifically bind HECT (Figure 3). This implies that the C-lobe truncation most likely folded into a similar structure as that in the context of the whole protein, consistent with the demonstrated activity of UbR5 [39]. Many ubiquitin ligases have been identified as disease targets. This is the first attempt to inhibit HECT ubiquitin ligase activity with an aptamer and we therefore do not have a direct comparison of binding affinity for this specific family of targets [40]. Aptamers often bind to targets with nanomolar affinities, but the *K*_D_ of our C3A could be estimated in the low µM range. We were not able to obtain estimations from our other aptamers and control because they did not bind sufficiently at the tested range of concentrations, but one may conclude their binding was far weaker (Figure 3). 

Ubiquitination assays to determine inhibition are well-established [41]. HECT ligases in particular are commonly studied from many angles with such assays [41]. Other groups have detected ubiquitination by anti-ubiquitin western blotting, but we found this step unnecessary given the quantitative Coomassie stain from the reaction product on the gel, which we confirmed to be ubiquitin conjugated HECT with mass spectrometry [40]. Nevertheless, IC_50_ values for aptamers are rarely reported because enzymes are typically targeted by small molecules. There are some examples, however, such as G-quadruplex aptamers against Shp2 phosphatase which inhibited Shp2 activity at 29 nM, while small molecules inhibited in the µM range [42]. Conversely, anti-sclerostin antibodies inhibit in the nM range, while aptamers for the same protein inhibit at 15 µM [19,43]. Evidence of the mode of inhibition could be seen with our Michaelis-Menten curves and apparent *v*_max_ and *K*_M_ values, which were consistent with non-competitive inhibition. Overall these pieces of evidence support the possibility that aptamer C3A binds specifically to HECT domain and inhibits its activity in a non-competitive manner (Figure 3 and Figure 4).

There are 3 known mechanisms for DNA to enter a eukaryotic cell without transfection or a virus: pinocytosis, absorptive endocytosis, or receptor mediated endocytosis [31,44]. In our assessment of aptamer C3A localization in SaOS-2 cells, we found qualitatively that the aptamer appeared to be able to enter the cell in the presence of absence of transfection reagent (Figure 5A). Here, we can only assume that the DNA in the non-transfected samples entered by the abovementioned mechanisms. Recently, aptamers for the intracellular and membrane target nucleolin were demonstrated to enter via macropinocytosis [45]. In addition, non-specific DNA internalizing receptors exist in other cell types but are not known in osteoblasts [27,28]. Both transfected and non-transfected samples were treated with DNA for 5 h, a time frame where aptamers could conceivably diffuse passively through the nuclear pore complex (Figure 5B), which could be compared to eukaryotic cells expressing proteins from plasmids which must enter the nucleus to be transcribed [29,46]. The percentage of signal appearing in the nucleus region for the hundreds of cells we analyzed with batch processing averaged around 60% (Figure 5C). One possible reason for this could be positively charged nuclear proteins such as histones, trapping a certain proportion of the aptamers.

Regarding aptamer C3A effect on phenotype, Western blot results were not clear-cut but were consistent with a slight reduction in Runx2 levels relative to controls (Figure 5D). The extracellular matrix deposition assay was performed under the same treatment conditions as the localization experiments and increased extracellular matrix was observed in the C3A treated cells relative to controls for both transfected and non-transfected cells.

Overall these experiments show that a DNA aptamer can bind specifically to WWP1 C-lobe and inhibit its target. Observations were consistent with the DNA aptamer entering the SaOS-2 cell nucleus, inhibiting WWP1 ubiquitination of Runx2 then increasing extracellular matrix deposition. WWP1 has also recently been implicated as an oncogene indicating that further work on WWP1 inhibition is warranted [47]. Finally, a recent paper demonstrated that interference of WWP1 led to the induction of apoptosis in osteosarcoma cells [47], which is also consistent with apoptosis experiments which we performed at the characterization stage (Figure S1). Further cell-based and animal-based experiments will be required to better understand the applicability of the aptamer to promote bone mineralization.

## 4. Materials and Methods 

### 4.1. HECT, C-Lobe, N-Lobe and E2 Cloning, Expression and Purification

HECT, C-lobe and N-lobe were amplified per Platinum^®^ Pfx Polymerase (Invitrogen, Waltham, MA, USA) guidelines from a human liver cDNA library. The following sequences were those for which the expression inserts were designed: HECT Domain (bp 1916–3047 NCBI Reference Sequence: NM_007013.3), C-lobe (bp 2687–3047 NCBI Reference Sequence: NM_007013.3), N-lobe (bp 2342–2587 in NCBI Reference Sequence: NM_007013.3). The resulting sequences, once inserted into pET28a(+) (Novagen, Madison, WI, USA) vector, gave an N-terminal hexahistidine tags. Subcloning was initiated by 1% agarose gel purification of the PCR product with Platinum^®^ Pfx Polymerase (Invitrogen) of each insert (approx 20 cycles) using QIAquick^®^ gel extraction kit (Qiagen, Hilden, Germany). Inserts were then digested with *Eco*RI and *Nde*I (New England Biolabs, Ipswich, MA, USA), 1% agarose gel purified and extracted, and the concentration of the insert and vector was then determined using absorbance at OD260/280 nm. Approximately 20 ng of insert and 10 ng of vector were put into ligation reaction with 1 µL ligase in total of 10 μL per instructions of T4 DNA ligase (New England Biolabs). A frozen eppendorf of XL-1 Blue *E. coli* (Stratagene, San Diego, CA, USA) provided in house was made competent and transformed by roughly following the pET System manual (Novagen). Colonies were selected on 50 µg/mL kanamycin agar plates, grown in 1 mL cultures for exponential growth phase, DNA was purified by QIAprep (Qiagen), and sequenced by Tech Dragon Limited™, Hong Kong, China.

The pET28a(+) (Novagen) vectors containing in-frame and un-mutated insert for all three truncations of HECT and pET-15b (Novagen) containing UbE2D2 (Addgene, Cambridge, MA, USA) were transformed into *E. coli* BL21(DE3) using the vendor transformation protocol (Novagen^®^). Overnight starter cultures of 30 µg/mL kanamycin containing LB media seeded 2–3 L cultures of the same media. These large cultures were grown at 37 °C under shaking at 200 rpm for approximately 6 h at 37 °C and induced to express with 500 µM IPTG, and shaken at room temperature for 4–5 h (C-lobe, N-lobe, and UbE2D2) and 4 °C overnight (HECT domain). Cell pellet was harvested by centrifugation at 4000 rpm, for 25 min, at 4 °C. After careful decanting, cell pellet was stored at −20 °C until purification. Column load was prepared by resuspending cell pellets in 20 mL of sonication buffer (Tris/500 mM NaCl/20 mM imidazole/0.1% (*v/v*) TritonX (pH 7.4)) for every 500 mL of culture and supplemented with Complete EDTA-free EASYpack protease inhibitor cocktail (Roche, Mannheim, Germany) followed by sonication for 10 min on ice at 30% amplitude. Cell lysate was centrifuged at 13,000 rpm for 25 min at 4 °C. Supernatant was filtered and loaded onto His-trap HP Ni^2+^ affinity column (GE Healthcare, Chicago, IL, USA) using a peristaltic pump. After loading, the column was transferred to Vision™ Workstation (Applied Biosystems, Waltham, MA, USA). Real time UV trace was analyzed with the accompanying Vision™ Software (SDS v. 2.3, Applied Biosystems, Foster City, CA, USA). A gradient was run from 0% mobile phase A (50 mM Tris/500 mM NaCl/20 mM Imidazole (pH 7.4)) → 100% mobile phase B (50 mM Tris/500 mM NaCl/500 mM Imidazole (pH 7.4)) over 20 min at 3.5 mL/min and ~2 mL fractions were taken by hand throughout the gradient. Fractions were stored at −20 °C. Overall, the proteins were of correct size, were derived from the correct sequence, had purities of around 95% (excluding UbE2D2), and maintained a solubility of around 50% of the starting fraction pool after one week at 4 °C. All proteins expressed in high yield (concentrations of 2–4 mg/mL) and signal during imidazole gradients generally coincided with predicted isoelectric points.

### 4.2. SELEX Procedures

Basic methodologies and tools for aptamer selection were adapted from our labs previous methods. A random DNA library was obtained from Tech Dragon Limited, Hong Kong containing 6.9 nanomoles of single stranded DNA of the following design: Aptamers→ 3’-CTAATACGACTCACTATAGG(N30)AAGCTTTGCAGAGAGGATCCTT-5’, Primers→ 3’-GATTATGCTGAGTGATATCC, TTCGAAACGTCTCTCCTAGGAA-5’-Biotin. DNA was reconstituted in MilliQ H_2_O and contained a total of 4.2 × 10^15^ DNA molecules of 1.15 × 10^18^ possible sequences from a variable region of *N* = 30. Determination of the amount of protein target to be used was based on the Ni-NTA Agarose Beads Handbook (Qiagen, Hilden, Germany) estimate that approximately 3 µg of protein bind to 10 µL of bead solution. The amount of target protein necessary to saturate 10 µL of bead solution was determined by exposing the beads to an increasing amount of protein, washing with selection buffer (50 mM Tris/0.05% Tween-20/0 → 1.0 M NaCl (pH 7.3)), eluting with 1.0 M imidazole in selection buffer, and visualizing on SDS-PAGE. After washing beads with protein buffer (50 mM Tris/400 mM NaCl/0.05% Tween-20 (pH 7.3)), appropriate amounts of protein were added and beads, and washed 3 times again with protein buffer. A 25 µL aliquot of library was then diluted with 200 µL of selection buffer and added to the beads, gently mixed, and set for 1 min. The beads were then washed with 1 mL of selection buffer 6–12 times depending on the round of selection. Protein bound to DNA was eluted with 50 µL elution buffer (1.0 M imidazole in TBS, pH 7.5) and 5 µL was carried on to PCR amplification with biotinylated reverse primer for ~10 cycles. The resulting double stranded amplification product from each consecutive round was separated by washing 50 µL of Dynabeads M-280 Streptavidin magnetic beads (Invitrogen, Waltham, MA, USA) with 1 mL separation buffer (50 mM Tris/0.05% Tween-20 (pH 7.3)), binding the entire 50 µL PCR product and 900 µL separation buffer with the washed beads, washing three times with separation buffer, and eluting with 50 µL of 100 mM NaOH. DNA containing NaOH was diluted with 150 µL TBS for neutralization. Progression of the selection process was monitored by PCR and PAGE. Pools of double stranded DNA from final rounds of selections were blunt end cloned into vectors using Zero Blunt^®^ TOPO^®^ PCR Cloning Kit (Invitrogen) and transfection of XL-1 Blue *E. coli* (Stratagene, San Diego, CA, USA) was performed as previously mentioned. 50 colonies from 50 µg/mL kanamycin selective plates were grown in 2.5 mL cultures for approximately 10 h, and said cultures were prepared for sequencing using the QIAprep^®^ Spin Miniprep Kit (Qiagen, Hilden, Germany). Sequencing of plasmids was performed by Tech Dragon Limited, Hong Kong, China.

### 4.3. HECT Ubiquitin Ligase Activity Assay 

A HECT ubiquitination assay was devised based on the general guidelines of the Boston Biochem^®^ Company which specializes in ubiquitin assays. Active HECT domain and the human E2 protein (UbE2D2) were expressed in *E. coli*, purified as described previously and were kept at 100 ng/µL and 160 ng/µL, respectively, at −20 °C in ~25% glycerol. His_6_-tagged human recombinant Ubiquitin and recombinant Human His_6_-Ubiquitin E1 Enzyme (UBE1) (Boston Biochem^®^) were kept at 5 µg/µL and 250 ng/µL, respectively, at −20 °C. The reaction was optimized by starting at 250 ng E1, 300 ng, HECT, 320 ng E2, and 5 µg Ub, and then systematically altering assay parameters such as reaction volume, time, reagent amounts, and temperature. Finally, the reaction contained 2.6 µM HECT, 45.5 nM UBE1, 507.8 nM UBE2D2, and varying from 300 nM to 4.8 µM Ubiquitin depending on the experiment. The total reaction volume was 25 µL, was run for 1 h at 37 °C in assay buffer (50 mM Tris/50 mM NaCl/5 mM MgCl_2_/5 mM KCl/25 mM DTT/5 mM ATP (pH 7.5)), and stopped by addition of 25 µL SDS-PAGE loading buffer (100 mM Tris/40% *v/v* glycerol/8% *w/v* SDS/5% *w/v* beta-mercaptoethanol/0.04% *w/v* bromophenol blue (pH 7.5)) with a final gel load of 20 µL. Identity of the reaction product band (HECT~Ub conjugate) was confirmed by MS/MS (Genome Research Center, Hong Kong, China). Gels were stained with Coomassie or Silver depending on need for quantitation and analyzed with ImageJ (NIH, Bethesda, MD, USA) and Prism^®^ (version 7, GraphPad, San Diego, CA, USA) softwares.

### 4.4. Electrophoretic Mobility Shift Assay (EMSA)

Briefly, 15 µL samples containing 11.3 µM → 293.9 nM HECT domain, 55.6 nM aptamer, and 4 µL of EMSA sample buffer (50 mM Tris/10% (*v/v*) Glycerol/0.02% (*w/v*) bromophenol blue (pH 6.8)) were created. The mixture was set for 60 min at 4 °C while gel was pre-run in TAE buffer at 100 V for equilibration. Samples were then loaded and run on 12% PAGE Gels which were prepared using vendor Midi Protean Cell guidelines (BioRad, Hercules, CA, USA) at 100 V for approximately 3 h until tracking dye reached bottom of the gel. Gels were stained with 3.5 µL of SYBR Gold Nucleic Acid Gel Stain (Molecular Probes, Waltham, MA, USA) per 50 mL of TAE buffer and imaged in the UV Transilluminator Imaging System (UVP, Hercules, CA, USA) and analyzed with ImageJ and Prism^®^. 6.5 pmol of aptamer was mixed with a series of concentrations of HECT, set for 1 h at 4 °C, run on 10% PAGE for 1.5 h at 100 V in 4 °C with minimal glycerol for gel loading, and stained with SYBR gold™.

### 4.5. Cell Culturing

SaOS-2 Cells (ATCC^®^ number HTB-85^®^), a human osteosarcoma line, were obtained in house labeled at passage 5. Cells were thawed, seeded, passaged according to the general guidelines of the Cell Culture Basics guide (Thermo Fisher Scientific, Hercules, CA, USA) in a Class II bio safety cabinet with McCoy’s 5a Media (Sigma-Aldrich, St. Louis, MO, USA) supplemented with Penicillin/Streptomycin and 15% FBS, and centrifugation was performed on bench top centrifuge. Cells were stored at a controlled environment of 37 °C with 5% CO_2_ and viewed with an Eclipse TS100 (Nikon, Tokyo, Japan) light microscope at 4× and 10× magnifications daily. Samples to be prepared for fixed cell imaging, western blot, apoptosis, bone mineralization, and alkaline phosphatase activity were initiated by growing cells to ~80% confluency on either 6, 24, or 96 well cell culture plates (Corning Inc., Corning, NY, USA). Cells were transfected with 100 ng of aptamer and control for 4 h per Lipofectamine 2000 (Invitrogen) guidelines using McCoy’s 5a Media (Sigma-Aldrich) without Penicillin/Streptomycin or FBS or treated with aptamer and control without transfection reagent for the same amount of time. Cells were then replaced with normal supplemented media after transfection, which took place once (T0) for fixed cell imaging, western blot, and apoptosis assays and three times (T0 then every 3 days) for mineralization and alkaline phosphatase assays. The cells remained alive for the course of the experiments.

### 4.6. Fixed Cell Imaging

Cover slip containing Costar^®^ 6 well flat bottom plates (Corning Inc., Corning, NY, USA) were prepared with sterile technique by placing cover slips into the wells with forceps in the BSC hood, rinsing with cold methanol, and rinsing 3× with ice cold sterile PBS. Cells were seeded 1:4 and grown to ~90% confluency and then treated with aptamer and controls, with and without Lipofectamine™ 2000. At each time point, wells were washed with ice cold PBS, gently fixed with ice cold methanol at −20 °C for one hour and washed with ice cold PBS. After damping dry, Acrytol^®^ mounting media (Leica, Wetzlar, Germany) was used to mount slides, which were then dried overnight in the dark. Images were taken on an BX51 Fluorescence Microscope (Olympus, Tokyo, Japan) using brightfield and the appropriate filters for Cy3 (red) and Hoechst (blue) at 40×. MetaMorph^®^ (Molecular Devices, San Jose, CA, USA) batch processing software was used to analyze at least 85 cells per sample.

### 4.7. Runx2 Western Blot

Cells were first washed with ice cold PBS, then 200 µL of ice cold RIPA buffer (150 mM sodium chloride/1.0% Triton X-100/0.5% sodium deoxycholate/0.1% SDS (sodium dodecyl sulfate)/50 mM Tris (pH 8.0)) supplemented with complete EDTA-free EASYpack protease inhibitor cocktail was added and cells, scraped and recovered by pipette. Lysates were agitated at 4 °C for 30 min by light shaking and centrifuged for 20 min at 12,000 rpm on a X-15R bench top centrifuge (Beckman Coulter) at 4 °C before recovering supernatant. In this case, Rabbit derived Anti-Runx2 (Novus Biologicals, Littleton, CO, USA) and Goat derived Anti-Rabbit IgG (whole molecule) Peroxidase conjugate (Novus Biologicals^®^) were used at 1/500 and 1/80,000, respectively, while generally following the Western Blotting Beginners Guide (Abcam, Cambridge, UK). 1 µL of B-Actin (Cell Signaling Technology, Danvers, MA, USA) stock was included with each primary antibody incubation as a loading control. Blots were imaged with UV Transilluminator Imaging System (UVP, Hercules, CA, USA).

### 4.8. Apoptosis Assay

Apoptosis samples were prepared as described in the cell culture section and were performed according to HT TiterTACS^™^ Assay kit (Trevigen, Gaithersburg, MD, USA) guidelines, read at 450 nm on a 200 SpectraMax 340PC 38 (Molecular Devices) and analyzed with Soft Max Pro (Molecular Devices) and Prism^®^ (GraphPad) Software.

### 4.9. Bone Mineralization Assay

Assays to detect the calcium of bone deposition generally followed the Osteoblast Differentiation and Mineralization Guide (PromoCell, Heidelberg, Germany). First, cells washed with ice cold PBS, fixed with ice cold methanol overnight, stained with 20 g/mL Alizarin Red S for 45 min at room temperature, washed 5 times with diH_2_O, and PBS added before digital photographs were taken.

## 5. Conclusions

We successfully generated a DNA aptamer (C3A) which binds to the C-lobe of WWP1 with a binding affinity of 1.9 μM. This aptamer was demonstrated to inhibit the ubiquitination activity of WWP1 in a non-competitive manner. The aptamer could internalize into SaOS2 cells even in absence of transfection agent. The aptamer was shown to stimulate extracellular matrix deposition relative to controls. Future work can improve binding affinity by microarray maturation, employ bioinformatics methods to modify the aptamer for greater functionality [48] or generate a library that could be used alongside a microfluidic selection method [49]. By such approaches, novel therapeutic aptamers can be further developed for a variety of human disease.

## Supplementary Materials

Supplementary materials can be found at http://www.mdpi.com/1422-0067/19/3/763/s1.
