# Supplementary material for: Selection and Characterization of a DNA Aptamer Specifically Targeting Human HECT Ubiquitin Ligase WWP1
Source: Int J Mol Sci. 2018 Mar 7;19(3):763. doi: 10.3390/ijms19030763 (PMC5877624; doi:10.3390/ijms19030763)
Supplement: Supplementary file 1 [file ijms-19-00763-s001.pdf]

Supplementary Materials: Selection and Characterization of a DNA Aptamer Specifically Targeting Human HECT Ubiquitin Ligase WWP1

Wesley O. Tucker, Andrew B. Kinghorn, Lewis A. Fraser, Yee-Wai Cheung, and Julian A. Tanner

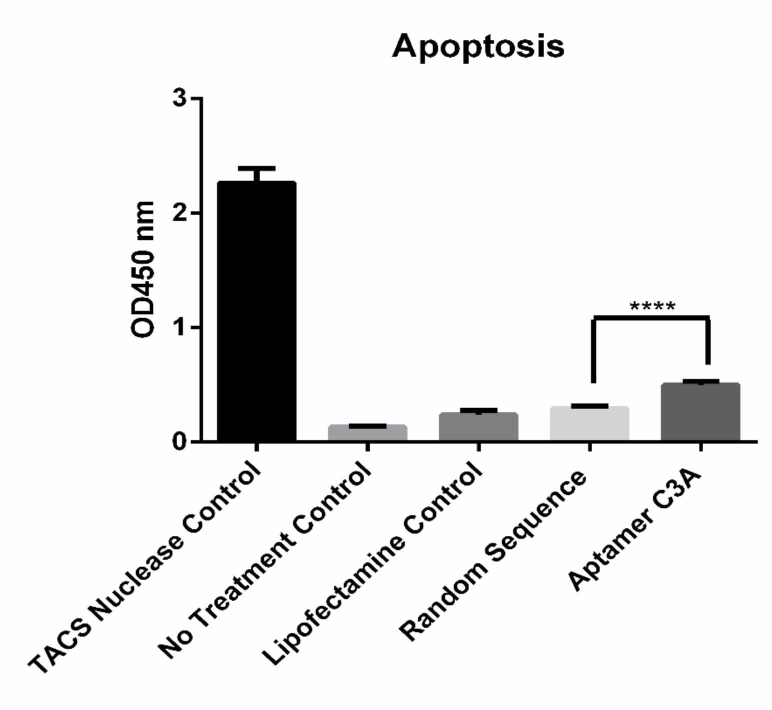

Figure S1. HT TiterTACS™ Assay kit (Trevigen®) apoptosis assay performed on SaOS-2 osteoblastic cells. The aptamer C3A was compared to TACS Nuclease positive control and no treatment, lipofectamine and random sequence controls.
